# In Situ Evaluation of the GSH Depletion Ability of Various Alkylating Agents and the Protective Effect of Several Active Thiol Compounds Based on High-Content Cell Analysis

**DOI:** 10.3390/toxics13121016

**Published:** 2025-11-24

**Authors:** Jing Guo, Zhi Li, Jiao Wang, Bo Ma, Liang Zhang, Hairui Wang, Jianfeng Wu, Jianwei Xie

**Affiliations:** 1 Academy of Military Medical Sciences, Beijing 100850, China; 2 Key Laboratory of Preparation and Applications of Environmental Friendly Materials, Ministry of Education, Jilin Normal University, Changchun 130103, China; 3 College of Resources and Environment, University of Chinese Academy of Sciences, Beijing 100049, China; 4 School of Pharmacy, Henan University, Kaifeng 475004, China

**Keywords:** GSH, alkylating agent, high-content cell analysis system, cytotoxicity, oxidative stress

## Abstract

The depletion degree of reduced glutathione is a critical indicator for assessing the toxicity of alkylating agents. In the present research, we have developed a novel method to evaluate the glutathione (GSH) depletion induced by a series of alkylating agents and the protective effect of various active thiol compounds based on a high-content cell analysis system. The cytotoxicity of some alkylating agents was first assessed using the CCK-8 assay. The results showed that bis(2-Choroethyl) methylamine (nitrogen mustard, HN2) and 1,2-bis(2-chloroethythio) ethane (Q) exhibited the highest cytotoxicity, with IC_50_ values of 14.45 μM and 23.27 μM, respectively. The cytotoxicity of 2-choroethylchoromethylsufide (CECM) and bis(2-choroethylthioethyl) ether (T) was comparable to that of bis(2-choroethyl) sulfide (HD), and bis(2-choroethylthiomethyl) ether (CEMEE) showed the lowest cytotoxicity. At the same exposure dose, Q exhibited the strongest GSH depletion ability, followed by HD > CECM > CEPR(1,3-bis(2-Chloroethylthio)-n-propane) > CEBU(1,4-bis(2-Chloroethylthio)-n-butane) > CEPE(1,5-bis(2-Chloroethylthio)-n-pentane) > CEME(bis(2-Chloroethylthio) methane) > T(bis(2-Choroethylthioethyl) ether) > CEMEE, and the depletion ability of nitrogen mustard compounds followed the order HN2 > HN1(bis(2-Choroethyl) ethylamine) > HN3(tri(2-Choroethyl) amine). In addition, the protective effect of four active thiol compounds was investigated. The results revealed that reduced glutathione ethyl ester (GSH-MEE) was most effective in preventing GSH depletion, whereas glutathione monoethyl ester (MEE) showed the highest efficacy in restoring GSH levels. The proposed method holds significant potential for analyzing the damaging effects of various alkylating agents and screening protective drugs.

## 1. Introduction

Glutathione (GSH), a highly active thiol tripeptide composed of glutamic acid, cysteine, and glycine, is present in nearly all cells and plays a crucial role in various biological processes. GSH not only maintains cellular redox balance and participates in antioxidant reactions but also regulates cell proliferation, immune responses, and acts as a neuromodulator and neurotransmitter in the nervous system [1,2,3,4,5].

As an important barrier against damage from exogenous alkylating agents, GSH plays a critical role in detoxifying exogenous chemicals and eliminating excessive reactive oxygen species (ROS) [6]. Beyond its significance in drug development, disease diagnosis, health monitoring and other research work [7,8], GSH levels serve as a key indicator of the antioxidant potential of the body [9]. According to research, normal cells typically contain GSH concentrations ranging from 0.5 to 10 mM [10]. However, exposure to alkylating agents, cancer, and oxidative stress can lead to abnormal fluctuations in GSH levels. For instance, vesicant chemical warfare agents, such as sulfur or nitrogen mustard [11,12,13], directly react with the sulfhydryl group of GSH [14], resulting in the accumulation of ROS and reactive nitrogen species, disruption of calcium homeostasis, lipid peroxidation, cell membrane rupture, and ultimately cell death. Additionally, under radiation exposure, the downregulation of superoxide dismutase (SOD) expression in hepatocytes, aimed at eliminating excess free radicals, results in the depletion of endogenous GSH [15]. Conversely, GSH levels in cancer cells increase following tumor formation. Therefore, monitoring and regulating GSH levels are crucial for disease prevention and treatment [16].

Numerous methods have been developed for GSH detection, including ultra-high-performance liquid chromatography (HPLC) [17], enzyme-linked immunosorbent assay (ELISA) [18], fluorescence spectroscopy [19], electrochemistry analysis [20], capillary electrophoresis [21], surface-assisted laser desorption ionization mass spectrometry (SALDI-MS) [22], and surface-enhanced Raman spectroscopy [23,24]. Among them, the spectrophotometric method based on the special color reaction of glutathione with 2-nitrobenzoic acid (DTNB) is both simple and sensitive. This method has been commercialized in kits, widely used for the rapid quantification of GSH in biological samples, including blood, urine, cells, and tissues [25]. A more accurate method involves the use of N-ethylmaleimide (NEM) to block sulfhydryl groups, combined with high-performance liquid chromatography (HPLC) to specifically label and quantify GSH. However, this method requires complex sample pretreatment and has a long detection time per sample [26]. Electrochemical methods offer advantages such as rapid response, low cost, simple operation, high sensitivity, and excellent selectivity. Ultra-sensitive electrochemical biosensors have been developed for the quantitative detection of GSH [27], but matrix interference remains a critical bottleneck that needs urgent resolution. To address the limitations of high-performance liquid chromatography (HPLC), a field detection method utilizing NaBH_4_ and surface-enhanced Raman spectroscopy (SERS) was developed. This method enables rapid evaluation of GSH depletion by alkylating agents, simplifies sample pretreatment, and significantly reduces detection time [28]. Although each of these technologies has its advantages, they all require sample destruction or disruption, precluding real-time, in situ, and nondestructive analysis.

The high-content cell analysis system (HCA) is an advanced screening technology that integrates high-resolution microscopic imaging, automated image collection, and complex biological sample analysis [29]. It can simultaneously detect various parameters, including cell morphology, growth, differentiation, migration, apoptosis, metabolic pathways, and signal transduction, while preserving cell structure and function integrity. HCA is widely used in toxicity testing, drug discovery, disease model research, 3D cell culture, and organ-like studies. Fluorescence analysis is a crucial component of HCA. Extensive research has focused on identifying specific cell structures or molecules and developing fluorescent probes with high sensitivity and specificity. For instance, Richter et al. used HCA technology to simultaneously monitor parameters such as the number of nuclei, mitochondrial membrane potential, cytoplasmic calcium levels, and cell membrane integrity, enabling the detection of cytotoxicity and metabolic characteristics of new psychoactive substances and other drugs of abuse in HepG_2_ cells, thereby improving the efficiency and information of toxicity tests [30]. Kim et al. utilized a high-content screening confocal microscope to offer a straightforward quantitative technique for evaluating GSH regeneration capability (GRC) [31]. HCA technology has been proven to exhibit high sensitivity and specificity in detecting toxic pathways induced by various drugs, particularly in predicting and evaluating drug-induced liver injury (DILI). To enhance the accuracy of clinical DILI evaluation in the drug screening process, Saito et al. developed an HCA analysis method to assess toxicity and predict drug-induced liver injury by detecting parameters such as ROS production, GSH consumption, and mitochondrial membrane potential (MMP) attenuation [32]. Pohan et al. investigated the assessment of drug-induced liver injury through multi-parameter high-content analysis and employed a variety of fluorescent dyes to monitor real-time cell health parameters associated with hepatotoxicity, including ATP levels, nuclear morphology, vacuole density, mitochondrial membrane potential, and ROS and GSH levels [33].

In this study, based on the characteristic structure of GSH, four probe molecules—RT-AM (a pro-agent real-thiol) [34], Mito RealThiol (MitoRT) [35], Thiol Tracker Violet [36], and monochlorodiamine (mBCL) [37]—were selected as the optimal probe molecule for GSH labeling in cells. To assess the cytotoxicity and GSH depletion capacity of 12 alkylating agents, a novel technique for real-time, nondestructive measurement of GSH levels in cells was developed by optimizing experimental parameters, such as probe concentration, incubation time, imaging conditions, and exposure dose. The technique is also employed to assess the therapeutic and preventive effects of several thiol compounds on traditional alkylating agents. The newly developed approach holds promising potential for studying toxicity mechanisms and evaluating drug safety.

## 2. Materials and Methods

### 2.1. Cell Culture

The human keratinocyte cell line HaCaT was provided by the Laboratory of Toxicant Analysis, Academy of Military Medical Sciences State, and purchased from ATCC. The HaCaT cells were cultured in DMEM medium (Gibco, Grand Island, NY, USA) supplemented with 10% fetal bovine serum (FBS) (Gibco, Grand Island, NY, USA), 100 units/mL penicillin and 100 μg/mL streptomycin (Gibco, Grand Island, NY, USA), and incubated at 37 °C in the presence of 5% CO_2_ in a humidified incubator.

### 2.2. Cytotoxicity Assay

Reference materials of nine sulfur mustard and three nitrogen mustard compounds, with purity greater than 95%, were provided by the PLA Army Chemical Defense College (Beijing, China). The chemical structures and physicochemical parameters are shown in Table S1. A series of alkylating agents with a concentration of 1 M were freshly prepared in dimethyl sulfoxide (DMSO), frozen and stored in the dark. They were reconfigured every month and diluted to varying concentrations (0–1000 μM) with serum-free DMEM medium immediately before the experiment. HaCaT cells were seeded into a 96-well culture plate at a density of 1 × 10^4^ cells/well and cultured in a 5% CO_2_ incubator at 37 °C for 24 h. The DMEM was removed, and the cells were exposed to a series of alkylating agents for 24 h. After exposure, CCK-8 reagent (Beyotime, Shanghai, China) was added to each well and incubated at 37 °C for 1 h. Absorbance at 450 nm was then measured using a microplate reader (Infinite M1000 pro, TECAN, Männedorf, Switzerland). The cell survival rate was calculated using the formula survival rate% = (A450 sample/A450 control) × 100%. GraphPad Prism software 10.1.2 was used to nonlinearly regress the dose–response curve, and the IC_50_ value was determined.

### 2.3. Fluorescent Probe Preferences

Four fluorescent probes specifically targeting GSH were selected to assess changes in intracellular GSH levels: RT-AM (MCE, Monmouth Junction, NJ, USA), MitoRT (Kerafast, Boston, MA, USA), Thiol Tracker Violet (Invitrogen, Carlsbad, CA, USA), and mBCL (Sigma-Aldrich, St. Louis, MO, USA). Stock solutions of each probe (1 M) were prepared in DMSO and stored in the dark. RT-AM, MitoRT, and mBCL solutions were diluted to final concentrations of 2.5 μM, 1 μM, and 20 μM, respectively, using serum-free DMEM medium. Thiol Tracker Violet solution was diluted to 20 μM with DPBS C/M buffer (Gibco, Grand Island, NY, USA). Subsequently, 100 μL of each probe solution was added to the cells and incubated for 30 min. The imaging performance of each probe was assessed using a high-content cell analysis system (Opera Phenix, PerkinElmer, Waltham, MA, USA). The excitation wavelengths for RT-AM and MitoRT were 405 nm and 488 nm, respectively, while Thiol Tracker Violet and mBCL were excited at 405 nm.

Common anions (Na^+^, SO_4_^2−^, NO_2_^−^), amino acids (NAC, Cys, L-His), and hydrogen peroxide (H_2_O_2_) obtained from Sigma-Aldrich (St. Louis, MO, USA) were used to evaluate the specificity of the fluorescent probes for GSH detection. The probe working solutions of the above concentrations were incubated with 1 mM GSH and 100 μM interferents for 15 min. Fluorescence intensity at the fixed excitation wavelength for each probe was measured using a microplate reader to assess the selectivity of the probes for GSH over other interferents. All incubations, including those with fluorescent probes, were carried out at 37 °C.

### 2.4. Optimization of Probe Incubation Concentration and Time

The optimal concentration and incubation time for the fluorescent probe mBCL were determined using a high-content cell analysis system. Cells were incubated with mBCL working solutions at concentrations ranging from 20 μM to 160 μM at 37 °C for 30 min to achieve specific GSH staining. The cells were washed three times with serum-free DMEM medium to remove unbound dye, and 100 μL of serum-free DMEM medium was added to each well. The plates were visualized and analyzed using a high-content cell imaging system to determine the optimal working concentration of mBCL. Subsequently, cells were incubated with the optimal mBCL concentration at 37 °C for 10 to 60 min at 10 min intervals. The optimal incubation time for mBCL was determined using the same procedure.

Additionally, N-ethylmaleimide (NEM; Sigma-Aldrich, St. Louis, MO, USA) was employed as a modulator to control intracellular GSH levels, allowing for the systematic alteration of GSH concentrations and the subsequent evaluation of mBCL as a fluorescent probe. The cell-seeded plates were treated with 10 μM NEM, stained with mBCL working solution, and incubated at 37 °C for 40 min. The plates were visualized, and changes in intracellular GSH levels were analyzed using a high-content cell imaging system.

### 2.5. Fluorescence Analysis of Intracellular GSH Changes

HaCaT cells were inoculated in 96-well culture plates at a density of 1 × 10^4^ cells/well and cultured at 37 °C in a 5% CO_2_ incubator. Cells were exposed to a series of alkylating agents at a concentration of 100 μM for 24 h. Control wells were supplemented with DMEM medium. After exposure, the cells were washed three times with PBS (Gibco, Grand Island, NY, USA). Cells were stained with a concentration of 100 μM mBCL (100 μL) and incubated for 40 min at 37 °C. Cell nuclei were stained with HCS NuclearMaskTM Deep Red (Invitrogen, Carlsbad, CA, USA) stain diluted to a 1× concentration. After staining, the cells were washed three times with serum-free DMEM medium, and the plates were visualized and analyzed using a high-content cell imaging system to assess the effect of each alkylating agent on intracellular GSH levels. Additionally, the effects of various alkylating agents on GSH level variations in cells were examined, along with changes in the mBCL-GSH complex with exposure time and dose.

### 2.6. Measurement of Reactive Oxygen Species Generation

Reactive oxygen species (ROS) levels in cells were measured using the fluorescent probe H2DCFDA reagent (Sigma-Aldrich, St. Louis, MO, USA). HaCaT cells were seeded into 96-well black cell culture plates at a density of 1 × 10^4^ cells/well. Before the experiment, a series of alkylating agents were diluted with serum-free DMEM medium to their final concentration, and the cells were exposed for 24 h. The H2DCFDA reagent was added to cells at a final concentration of 25 μM and incubated for 30 min at 37 °C. The medium was removed, and cells were washed three times with PBS (Gibco, Grand Island, NY, USA). The plates were visualized and analyzed using a high-content cell imaging system.

### 2.7. Screening of Protective Drugs and Optimization of Conditions

HaCaT cells were seeded in 96-well black cell culture plates at a density of 1 × 10^4^ cells/well. GSH, N-acetylcysteine (NAC), reduced glutathione ethyl ester (GSH-MEE) and glutathione monoethyl ester (MEE) obtained from Sigma-Aldrich (St. Louis, MO, USA) were dissolved in PBS. Immediately before the experiment, the compounds were continuously diluted with serum-free DMEM medium to a final concentration of 1 mM. The experiment was divided into two groups: in the first group, the cells were pretreated with 1 mM protective drugs for 6 h, washed three times with PBS and exposed to HD (120 μM) for 6 h; in the second group, cells were exposed to HD (120 μM) for 6 h, then washed three times with PBS, and treated with 1 mM protective drugs for 6 h. All groups included a control group, HD group, and protective drug-only exposure group. The mBCL was added to the cells at a final concentration of 100 μM and incubated at 37 °C for 40 min. The medium was removed and the cells were washed three times with PBS. The plates were visualized and analyzed using a high-content cell imaging system. At the same time, the concentration of the optimal prevention and recovery reagent was optimized using the same method, and the prevention and recovery effects of four classic alkylating agents—HD (120 μM), CECM (100 μM), Q (20 μM), and HN2 (10 μM)—were evaluated.

### 2.8. Statistical Analysis

Data are presented as the means ± standard deviation (SD) of three independent experiments. Statistical analyses were conducted using Student’s *t*-test or one-way analysis of variance (ANOVA), followed by Dunnett’s test with GraphPad Prism 8 software (* *p* < 0.05, ** *p* < 0.01, *** *p* < 0.001, **** *p* < 0.0001).

## 3. Results

### 3.1. Cytotoxicity Induced by Series of Alkylating Agents

**Figure 1:**
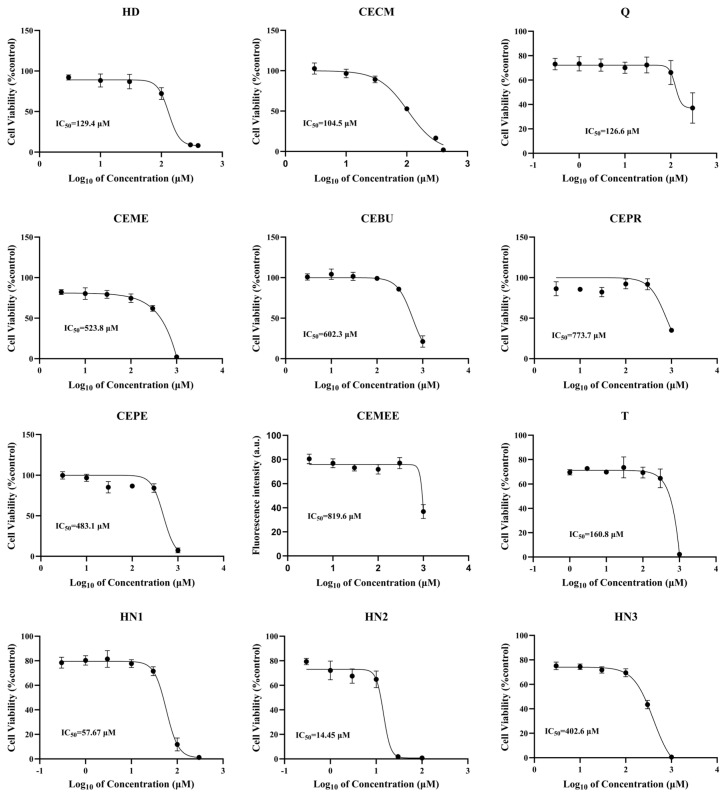
Cytotoxicity profile and IC_50_ values for the series of alkylating agents. HaCaT cells were treated with the chemicals at indicated concentrations, and cytotoxicity assays were performed 24 h after treatment. Data represent the mean (±SD) of 5–6.

To determine the optimal concentration of each alkylating agent, the cytotoxicity of various alkylating agents was assessed using the human immortalized keratinocytes cell line HaCaT as a model [38]. As exhibited in Figure 1, all nine sulfur mustard compounds showed dose-dependent cytotoxicity. Consistent with previously published results [11], the IC_50_ value of HD is 129.4 μM, while Q and CECM exhibit greater cytotoxicity than HD, with IC_50_ values of 23.27 μM and 104.5 μM, respectively. The cytotoxicity of three nitrogen mustard compounds followed the order HN2 > HN1 > HN3, with their IC_50_ values of 14.45 μM, 57.67 μM, and 402.6 μM, respectively. Based on the above results, the optimal concentration of each alkylating agent was determined based on its IC_50_ value.

### 3.2. Preferred Specific Recognition of GSH Fluorescent Probes

**Figure 2:**
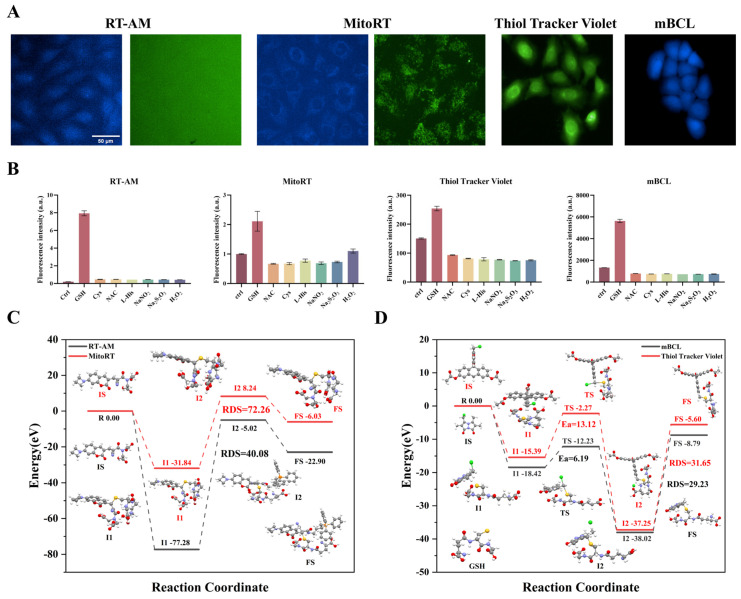
(**A**) High-content cell imaging of each fluorescent probe: RT-AM and MitoRT are both ratio fluorescent probes, with an excitation wavelength of 405 nm for RT-AM and MitoRT and a channel color of blue for RT-AM and MitoRT, and a wavelength of 488 nm for the adduct with GSH, with a channel color of green. (**B**) Specificity of each fluorescent probe: RT-AM and MitoRT can detect a change in F405/F488 when they react with GSH, while Thiol Tracker Violet and mBCL can detect a change in fluorescence intensity at the excitation wavelength of 405 nm. DFT calculation, reaction process and free energy change of four probes with GSH: (**C**) RT-AM and MitoRT, (**D**) mBCL and Thiol Tracker Violet.

To accurately track the dynamic changes of GSH in cells, four fluorescent probes—RT-AM, Mito-RT, Thiol Tracker Violet, and mBCL—were selected based on reported literature. Among these, RT-AM exhibits good specificity and can quantitatively monitor dynamic changes in GSH in living cells in real time. Mito-RT can dynamically monitor changes in mitochondrial-specific GSH. Thiol Tracker Violet has a fast response time and high sensitivity to thiols. MBCL specifically binds to GSH to form fluorescent complexes. The structural formulas of the probes are shown in Figure S1. After 30 min of incubation with cells, the imaging quality and specificity of the four fluorescent probes were evaluated using an enzyme-labeled analyzer and a high-content cell analysis system. The excitation wavelengths of RT-AM, Mito-RT, and their GSH adducts are 405 and 488 nm, respectively. When they react with GSH, they exhibit a broad dynamic fluorescence response. Figure 2A shows the fluorescence imaging results. At an excitation wavelength of 488 nm, the fluorescence in cells is barely visible, and the RT-AM imaging effect is poor. The fluorescence imaging of cells remains unclear. Mito-RT is a fluorescent probe that targets GSH in mitochondria. At an excitation wavelength of 488 nm, the mitochondrial boundaries are unclear. Compared to RT-AM and Mito-RT, the imaging effects of the Thiol Tracker Violet and mBCL probes are significantly better.

Figure 2B illustrates the selectivity of each probe in a physiological environment. When RT-AM and Mito-RT react with GSH, the ratio of F405/F488 changes, but their readings specifically respond to GSH without interference from other substances. Other thiols or active nitrogen compounds do not exhibit any changes at their respective physiological concentrations. MBCL and Thiol Tracker Violet show a strong reaction to GSH. In contrast, mBCL exhibits a stronger reaction and is more selective for glutathione. Considering the quality and specificity of fluorescence imaging, mBCL was selected as the fluorescence probe to monitor changes in GSH levels in cells in subsequent studies.

At the same time, the density functional theory (DFT) is calculated by the Gauss 09 software package [39]. Ground-state optimization and transition-state search were calculated at the PBE0/6-31g (d, p) level for all atoms [40]. Vibrational frequency calculations at the same level were performed to verify that each stationary point reached a minimum (no imaginary frequency) or a transition state (only one imaginary frequency). The dispersion corrections with Grimme’s D3 method [41] were taken into consideration in all calculations. As shown in Figure 2C,D, the four fluorescent probes reacted with GSH under the same conditions, and the activation energy of mBCL in RDS was the smallest, which was 29.23 kJ/mol, indicating that mBCL is most likely to react with GSH, further confirming the accuracy of the selected fluorescent probes.

### 3.3. Optimization of Imaging Conditions for Fluorescent Probes

**Figure 3:**
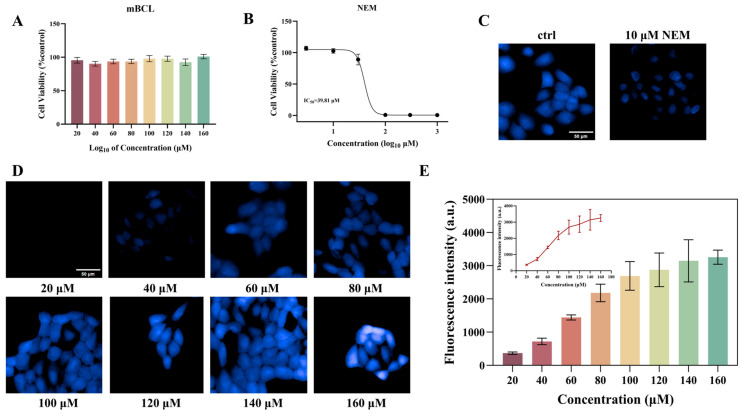
(**A**) mBCL cytotoxicity test (2 h); (**B**) NEM cytotoxicity test (24 h); (**C**) co-incubating 10 μM NEM with cells for high-content cell imaging; (**D**) high-content cell imaging with varying concentrations of mBCL; Scale bars: 50 μm. (**E**) fluorescence intensity analysis following incubation with cells of various cocentrations. Data are expressed as mean ± SD of 5–6.

To determine the optimal application concentration of the fluorescent probe mBCL and GSH consumption agent NEM, their cytotoxicity was assessed using the CCK-8 method. After 2 h of incubation with HaCaT cells, the cell survival rate remained above 80% (Figure 3A), indicating that mBCL did not exhibit discernible cytotoxicity to HaCaT cells within this concentration range. The IC_50_ value of NEM is 39.81 μM (Figure 3B). On the other hand, NEM maintained more than 95% cell viability at a low concentration of 10 μM. When HaCaT cells were co-incubated with 10 μM NEM for 24 h and stained with mBCL dye, a significant decrease in fluorescence intensity was observed using high-content cellular imaging (Figure 3D). Additionally, after co-incubation, cells from eight different concentrations were selected for high-content cell imaging and data analysis. A clear trend was observed in the fluorescence intensity and data analysis. As the concentration of mBCL increased, the fluorescence intensity gradually intensified, indicating that mBCL could effectively react with intracellular GSH to generate fluorescence signals (Figure 3E). When the concentration of mBCL reached 100–120 μM, the increase in fluorescence intensity plateaued (Figure 3E), indicating that the staining efficiency of mBCL approached saturation within this concentration range. Therefore, mBCL can be used to evaluate dynamic changes in GSH levels in cells, and its optimal incubation concentration is 100 μM.

The incubation time between the fluorescent probe and cells is another crucial factor affecting imaging quality. mBCL at 100 μM was incubated with cells for 10, 20, 30, 40, 50, and 60 min, respectively. The results showed that fluorescence intensity increased rapidly within the 10–40 min range. Extending the incubation time to 60 min caused the fluorescence intensity to increase at a slower rate. Considering both fluorescence intensity and imaging quality, we determined that the optimal incubation time for mBCL was 40 min (Figure S2).

### 3.4. Evaluation of Different Alkylating Agents on the Capacity of Intracellular GSH Depletion

**Figure 4:**
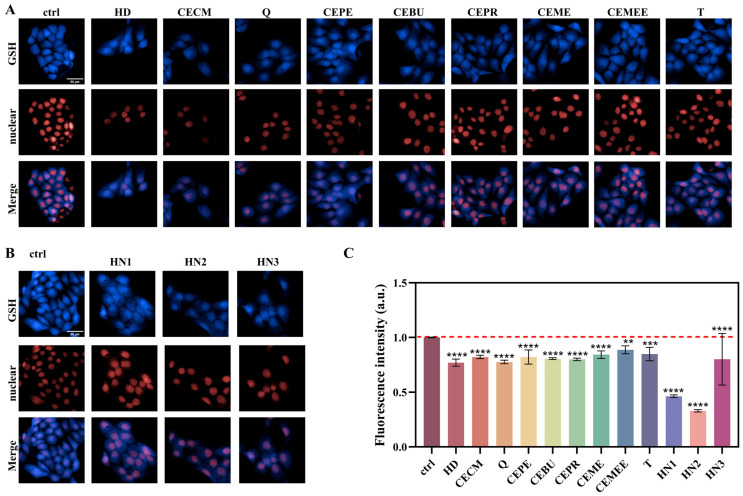
Series of alkylating agent-induced intracellular GSH depletion in HaCaT cells, visualized by HCS system imaging and analysis. (**A**) High-content cell imaging of nine sulfur mustard-induced changes in intracellular GSH levels. (**B**) High-content cell imaging of three nitrogen mustard-induced changes in intracellular GSH levels. Scale bars: 50 μm. (**C**) Evaluation of fluorescence intensities of the cells with the HCS system. Data are expressed as mean ± SD of 5–6. Statistical analyses were performed using the one-way ANOVA test followed by Dunnett’s multiple comparison test, where ** *p* < 0.01, *** *p* < 0.001 and **** *p* < 0.0001 indicate the level of statistical significance.

A key mechanism underlying the cytotoxicity induced by alkylating agents is GSH depletion. The subsequent toxic effects vary due to differences in molecular structure, composition, and active functional groups among the various alkylating agents. Under the optimal experimental conditions outlined above, the GSH consumption capacity of nine sulfur mustard compounds and three nitrogen mustard compounds was assessed. The results are shown in Figure 4 that fluorescence intensities of alkylating agent-treated cells exhibited varying degrees of reduction compared to the control group, with the three nitrogen mustards causing a more significant reduction in fluorescence intensity than the nine sulfur mustards. Among the nine sulfur mustards, Q exhibits the strongest GSH depletion ability, followed by HD and CECM. Five sesqui-mustard compounds exhibit stronger GSH depletion ability than the two oxy-mustard compounds, with Q showing the strongest depletion ability among the five sesqui-mustards, which is consistent with the cytotoxicity data of different sulfur mustard compounds mentioned above. The GSH depletion ability of the three nitrogen mustards follows the order HN2 > HN1 > HN3. HN2 exhibits strong alkylation ability, making it more reactive in cells, thereby enhancing its toxicity and GSH consumption capacity.

### 3.5. Dose- and Time-Dependent Depletion of Intracellular GSH by Alkylating Agent

**Figure 5:**
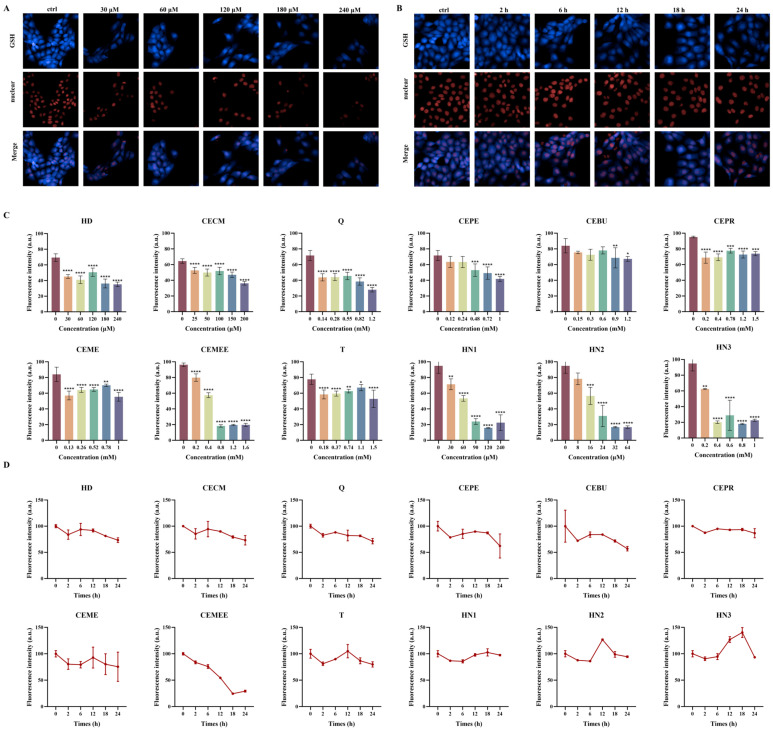
Sulfur mustard and nitrogen mustard induced GSH depletion in HaCaT cells in a dose- and time-dependent manner. (**A**) Dose–response relationship with GSH depletion formation after HD treatment evaluated by high-content cell analysis system. (**B**) Time–response relationship with GSH depletion formation after Q treatment evaluated by high-content cell analysis system. Scale bars: 50 μm. (**C**) Bar graph of the dose–response relationship with GSH depletion formation after the rest of the alkylating agent treatments. (**D**) Histogram of the time–response relationship with GSH depletion formation after the remaining alkylating agent treatments. Data are expressed as mean ± SD of 5–6. Statistical analyses were performed using the one-way ANOVA test followed by Dunnett’s multiple comparisons test, where * *p* < 0.05, ** *p* < 0.01, *** *p* < 0.001, and **** *p* < 0.0001 denote the level of statistical significance.

Although studies on GSH depletion induced by HD and nitrogen mustards have been reported, research on their analogues is limited, and the description of their depletion process remains insufficient. Therefore, the dose-dependent and time-dependent depletion relationship is evaluated. As shown in Figure 5A, taking HD as an example, the fluorescence intensity of HaCaT cells exhibited a decreasing trend after exposure. As the HD concentration increased, the fluorescence intensity increased when the exposure dose reached 120 μM. With further increases in HD concentration, fluorescence intensity continued to decrease, and the fluorescence intensity of other compounds showed similar trends (Figure 5C).

The depletion of GSH induced by alkylating agents triggers the intracellular antioxidant mechanism. The toxicity of different exogenous alkylating agents varies after exposure, leading to differing times for triggering self-regulation. As shown in Figure 5B, taking Q as an example, the fluorescence intensity of cells decreased after 2 h of exposure, increased after 6 h, and continued to decrease with further extension of exposure time. The changes in fluorescence intensity of other compounds are similar, but the timing for inducing cell self-regulation varies. As shown in Figure 5D, among the nine sulfur mustard compounds, exposure to CECM for 6 h triggers the ability to induce self-regulation, while exposure to CEME and T for 12 h triggers self-regulation and restores a certain level of glutathione. Among nitrogen mustard compounds, HN2 induces cell self-regulation after 12 h of exposure, while HN1 and HN3 trigger self-recovery over a longer period. Other alkylating agents induce GSH depletion in cells, with dose- and time-dependent fluorescence images triggering self-regulation shown in Figures S3 and S4.

### 3.6. Alkylating Agents Induce the Production of Reactive Oxygen Species

**Figure 6:**
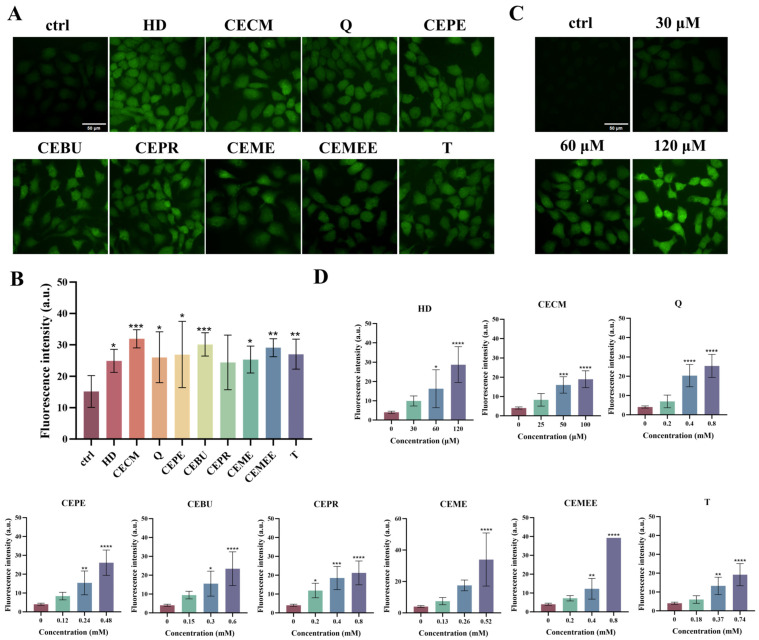
Sulfur mustard induces intracellular ROS production. (**A**) Evaluation of ROS content in cells induced by 9 sulfur mustards by high-content cell imaging. (**B**) Analysis of high-content cell imaging data. Scale bars: 50 μm. (**C**) High-content cell imaging to evaluate the dose relationship of the increase in intracellular ROS content induced by HD. (**D**) Histogram of the dose relationship of 9 sulfur mustard-induced increases in intracellular ROS content. Data are expressed as mean ± SD of 5–6. Statistical analyses were performed using the one-way ANOVA test followed by Dunnett’s multiple comparisons test, where * *p* < 0.05, ** *p* < 0.01, *** *p* < 0.001, and **** *p* < 0.0001 denote the level of statistical significance.

GSH, as a key cellular antioxidant, plays a crucial role in maintaining the cell’s antioxidant defense mechanism. The depletion of GSH not only weakens the cell’s antioxidant capacity but also leads to oxidative stress-related damage. To explore the mechanism of oxidative stress induced by alkylating agents and their potential applications, we compared ROS production in HaCaT cells treated with alkylating agents and control cells. High-content cell imaging data revealed that cells treated with alkylating agents showed a significant increase in green fluorescence intensity compared to the untreated control group (Figure 6A,B). This increase became more pronounced with higher exposure concentrations of alkylating agents (Figure 6C,D).

### 3.7. Evaluation of the Antagonistic Effect of Different Exogenous GSH Modulators

Alkylating agents cause GSH depletion, which in turn induces toxic effects such as oxidative stress, DNA damage, and ferroptosis. Therefore, when the body is exposed to exogenous substances, the level of GSH may fail to self-regulate and return to a normal state. Currently, various countermeasures against GSH depletion include direct supplementation of GSH and its derivatives [42,43], activation of key enzymes involved in GSH synthesis [44], addition of antioxidants, and enhancement of GSH synthesis and gene expression via gene transfer technology [45]. Among these, direct application of GSH or its derivatives is the simplest, most effective, and most commonly used countermeasure.

**Figure 7:**
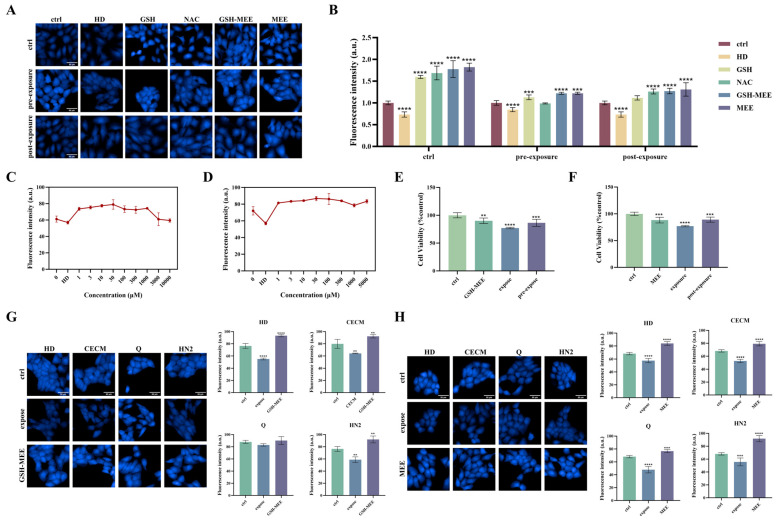
(**A**) Screening four kinds of protective drugs for the high-content cell analysis system. Control group: cells incubated with solutions containing 1 mM protective drugs for 12 h; pre-exposure group: cells pretreated with 1 mM protective drugs for 6 h and exposed to HD (120 μM) for 6 h; post-exposure group: cells exposed to HD (120 μM) for 6 h, and 1 mM protective drugs for 6 h; control group: cells exposed to alkylating agent and mercaptan compounds. Scale bars: 50 μm. (**B**) Analyzing the high-content imaging data. (**C**) Optimizing the incubation concentration of GSH-MEE. (**D**) Optimal incubation concentration of MEE. (**E**) Cell viability determination: cells were pretreated with 30 μM GSH-MEE for 6 h and exposed to HD (120 μM) for 6 h. (**F**) Cell viability determination: after the cells were exposed to HD (120 μM) for 6 h, they were treated with 30 μM MEE for 6 h. (**G**) Evaluation of the preventive effects of GSH-MEE against four alkylating agents. (**H**) Evaluation of the recovery effect of MEE on four classical alkylating agents. Data are expressed as mean ± SD of 5–6. The statistical analysis was conducted using the single-component ANOVA test and the Dunnett multiple comparison test, where the degree of statistical significance is indicated by ** *p* < 0.01, *** *p* < 0.001, and **** *p* < 0.0001.

Using the established high-content technology, we screened four protective drugs—GSH, NAC, GSH-MEE, and MEE—and investigated their preventive and restorative effects against HD (120 μM). The results (Figure 7A,B) show that the four protective drugs effectively increase the GSH level in cells. Among these, GSH-MEE exhibits the most significant preventive effect, while MEE demonstrates the most pronounced recovery effect. Additionally, the culture concentration was optimized. The results showed that fluorescence intensity gradually increased with concentration and peaked at 30 μM (Figure 7C,D). When the concentration exceeded this value, fluorescence intensity decreased. Therefore, we selected 30 μM as the optimal concentration, as it results in the maximum intracellular glutathione level.

Meanwhile, the cell activity before and after protection was assessed. The results showed that cell viability following treatment with GSH-MEE and MEE was significantly lower than that of the cells treated with HD alone, indicating that both effectively reduce HD cytotoxicity and provide clear protective effects (Figure 7E,F). Furthermore, the prevention and recovery effects of GSH depletion caused by exposure to HD (120 μM), CECM (100 μM), Q (20 μM), and HN2 (10 μM) were evaluated. As seen in Figure 7E,F, after adding GSH-MEE or MEE, the fluorescence intensity in cells significantly increased, indicating an increase in intracellular GSH levels. In contrast, exposure to alkylating agents led to a significant decrease in intracellular GSH levels, highlighting GSH’s crucial role in preventing depletion caused by alkylating agents.

## 4. Discussion

The aim of this study was to develop a novel method for the rapid and nondestructive determination of intracellular GSH levels, assess the GSH depletion capacity of various alkylating agents, and investigate the preventive and restorative effects of protective drugs against alkylating agent-induced GSH depletion. Intracellular GSH levels were successfully monitored in real time through the screening of fluorescent probes and optimization of incubation conditions. The dose- and time-dependent effects of alkylating agents on GSH depletion were thoroughly investigated, confirming the regulation of intracellular antioxidant mechanisms and the presence of oxidative stress. Furthermore, thiol compounds effectively mitigated GSH depletion induced by alkylating agents and demonstrated significant preventive and therapeutic potential.

Epidermal keratinocytes are considered one of the main targets of sulfur mustard- and nitrogen mustard-induced pathology, and HaCaT cells are used as models to assess the cytotoxicity of various alkylating agents and determine the optimal exposure concentration. Alkylating agents exhibited cytotoxicity in a dose-dependent manner. Specifically, we confirmed that the cytotoxicity of Q and CECM was stronger than that of HD, while the cytotoxicity of T was similar to that of HD. The cytotoxicity of nitrogen mustard compounds follows the order HN2 > HN1 > HN3 (Figure 1), consistent with the toxicity data reported by Laskin, J D. et al. [12].

In this study, mBCL, a fluorescent probe specifically designed to recognize GSH, was selected, and its optimal incubation conditions were determined. Experimental results indicate that the optimal incubation concentration of mBCL is 100 μM (Figure 3). At this concentration, the binding efficiency between the probe and intracellular GSH is maximized, and the fluorescence intensity peaks, accurately reflecting the intracellular GSH level. At this concentration, mBCL also determined that the optimal incubation time was 40 min (Figure S2).

Due to differences in the chemical structure and active groups of various alkylating agents, their ability to deplete GSH and cause toxicity in cells varies. We have demonstrated that the ability of various alkylating agents to induce GSH depletion is significantly different. Among them, nitrogen mustards are more likely to form highly reactive onium intermediates, which can react more effectively with sulfur-containing molecules such as GSH in cells, resulting in more significant GSH depletion compared to the nine sulfur mustard species (Figure 4). Numerous studies have reported that exposure to HD significantly induces intracellular GSH depletion. The sulfhydryl reaction between alkylating agents and GSH leads to a decrease in its level, which subsequently induces a series of cellular injury responses, such as ROS accumulation, consistent with the mechanisms described in the literature. Experiments showed that alkylating agents react with GSH in cells after exposure, leading to a decrease in GSH levels. As GSH consumption and exposure concentration increase, the antioxidant capacity of cells decreases and ROS content increases, triggering oxidative stress and the cell’s self-regulation mechanism, which leads to increased GSH synthesis and rebounding fluorescence intensity. However, under prolonged high-concentration exposure to alkylating agents, the cells’ antioxidant capacity is damaged, and excessive ROS cannot be effectively cleared, leading to persistent oxidative stress and an inability to regenerate sufficient GSH to counter it, causing a continuous decline in GSH levels (as shown in Figure 5 and Figure 6).

Exposure to alkylating agents typically leads to GSH depletion, disrupting the redox balance of cells and inducing cellular damage and dysfunction. Therefore, screening effective drugs to protect against GSH depletion, alleviate oxidative stress damage, and elucidate their protective mechanisms is of great significance. The four protective drugs we screened are all thiol-based compounds. These compounds protect by competitively binding to alkylating agents with their sulfhydryl groups, reducing GSH consumption. We found that GSH-MEE exhibited the most significant preventive effect, while MEE demonstrated the most pronounced recovery effect on GSH depletion induced by alkylating agents. Both compounds effectively counteract the cytotoxicity of these agents. GSH-MEE and MEE, as ester derivatives of GSH, exhibit greater protective effects than GSH itself, likely due to their enhanced cell membrane permeability, allowing hydrolysis by intracellular enzymes and the release of GSH. The prodrug-like mechanism of these compounds not only reduces the required dosage and improves compound stability but also effectively mitigates the cytotoxicity of alkylating agents, offering a promising approach for treating alkylating agent-induced injury. The potential broader application of this method is evaluating the toxicity of other classes of GSH-consuming toxicants.

## 5. Conclusions

GSH is a crucial indicator for evaluating the cytotoxicity and damaging potential of various alkylating agents. Given the lack of real-time, nondestructive methods to monitor changes in cellular GSH levels, mBCL was selected from several classic fluorescent probes (e.g., RT-AM, MitoRT, Thiol Tracer Violet) to establish a novel method for in situ GSH evaluation using advanced cell imaging technology. The depletion capacity of 12 highly active alkylating agents was subsequently investigated in detail. Using the developed method, the counteracting effects of a series of exogenous GSH regulators were evaluated. Nitrogen mustard and certain sulfur mustards exhibit stronger GSH consumption abilities due to their higher chemical reactivity and enhanced cell penetration. Furthermore, GSH ester derivatives demonstrated lower cytotoxicity, required lower dosages, and provided better GSH regulation and recovery due to their improved cell permeability and stability. Compared to previous GSH detection methods, our newly established approach offers higher throughput, more intuitive results, and simpler discrimination. The key advantage is the ability to evaluate the GSH depletion capacity of highly toxic alkylating agents in situ and in real time, without compromising cell morphology or physiological state, while identifying the best candidate drugs. This method holds great potential for preventing and treating the harm caused by these alkylating agents or addressing sudden poisoning incidents.

## Data Availability

The original contributions presented in this study are included in the article/Supplementary Materials. Further inquiries can be directed to the corresponding authors.

## Supplementary Materials

The following supporting information can be downloaded at: https://www.mdpi.com/article/10.3390/toxics13121016/s1, Table S1. The chemical structural formula and physicochemical parameter information of alkylating agents. Figure S1. Structure of each fluorescent probe. Figure S2. High-content cell imaging and data analysis of mBCL incubated with cells for different times. Figure S3. High-content cell imaging of GSH depletion induced by a series of alkylating agents in HaCaT cells in a dose-dependent manner. Figure S4. High-content cell imaging of GSH depletion induced by a series of alkylating agents in HaCaT cells in a time-dependent manner.
