# Supplementary material for: In Situ Evaluation of the GSH Depletion Ability of Various Alkylating Agents and the Protective Effect of Several Active Thiol Compounds Based on High-Content Cell Analysis
Source: Toxics. 2025 Nov 24;13(12):1016. doi: 10.3390/toxics13121016 (PMC12737281; doi:10.3390/toxics13121016)
Supplement: Supplementary file 1 [file toxics-13-01016-s001.zip › toxics-3973885-supplementary.pdf]

Supporting information

In situ evaluation of the GSH depletion ability of various alkylating agents and the protective effect of several active thiol compounds based on high-content cell analysis system

Jing Guo [1, 2 †], Zhi Li [1, †], Jiao Wang [1, 3], Bo Ma [1], Liang Zhang [1,4], Hairui Wang [2], Jianfeng Wu [1 *], Jianwei Xie [1, *]

[1] *Academy of Military Medical Sciences, Beijing 100850, China*

[2] *Key Laboratory of Preparation and Applications of Environmental Friendly Materials, Ministry of Education, Jilin Normal University, Changchun 130103, China*

[3] *College of Resources and Environment, University of Chinese Academy of Sciences, Beijing 100049, China*

[4] *School of Pharmacy, Henan University, Kaifeng 475004, China*

Table S1. The chemical structural formula and physicochemical parameter information of series alkylating agents.

Compound	Structure	Molecular mass	CAS number
2-Chloroethylchoromethylsufide （CECM）	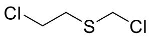	145.05	2625-76-5
bis(2-Chloroethy) sulfide （HD）	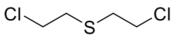	159.08	505-60-2
bis(2-Chloroethylthio) methane （CEME）	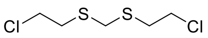	205.2	63869-13-6
1,2-bis(2-Chloroethylthio) ethane （Q）	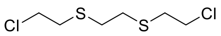	219.2	3563-36-8
1,3-bis(2-Chloroethylthio)-n-propane （CEPR）	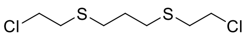	233.2	63905-10-2
1,4-bis(2-Chloroethylthio)-n-butane （CEBU）	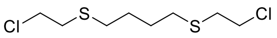	247.3	142868-93-7
1,5-bis(2-Chloroethylthio)-n-pentane （CEPE）	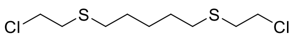	261.3	142868-94-8
bis(2-Choroethylthiomethyl) ether (CEMEE)	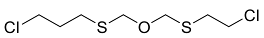	235.2	63918-90-1
bis(2-Choroethylthioethyl) ether （T）	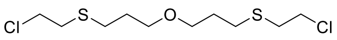	263.2	63918-89-8
bis(2-Choroethyl) ethylamine （HN1）	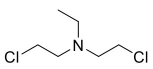	170.08	538-07-8
bis(2-Choroethyl) methylamine （HN2）	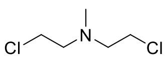	156.05	51-75-2

tri(2-Choroethyl) amine (HN3)	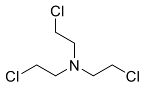	204.5	555-77-1

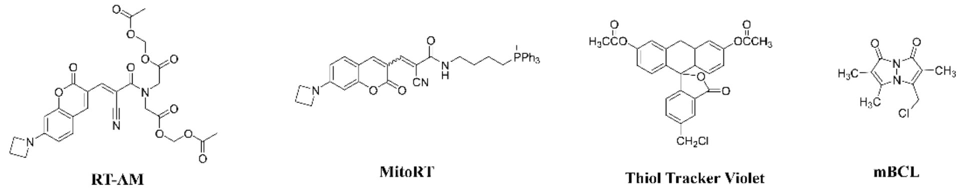

Figure S1 Structural of each fluorescent probe.

Figure S2 High-content cell imaging and data analysis of mBCL incubated with cells for different time.

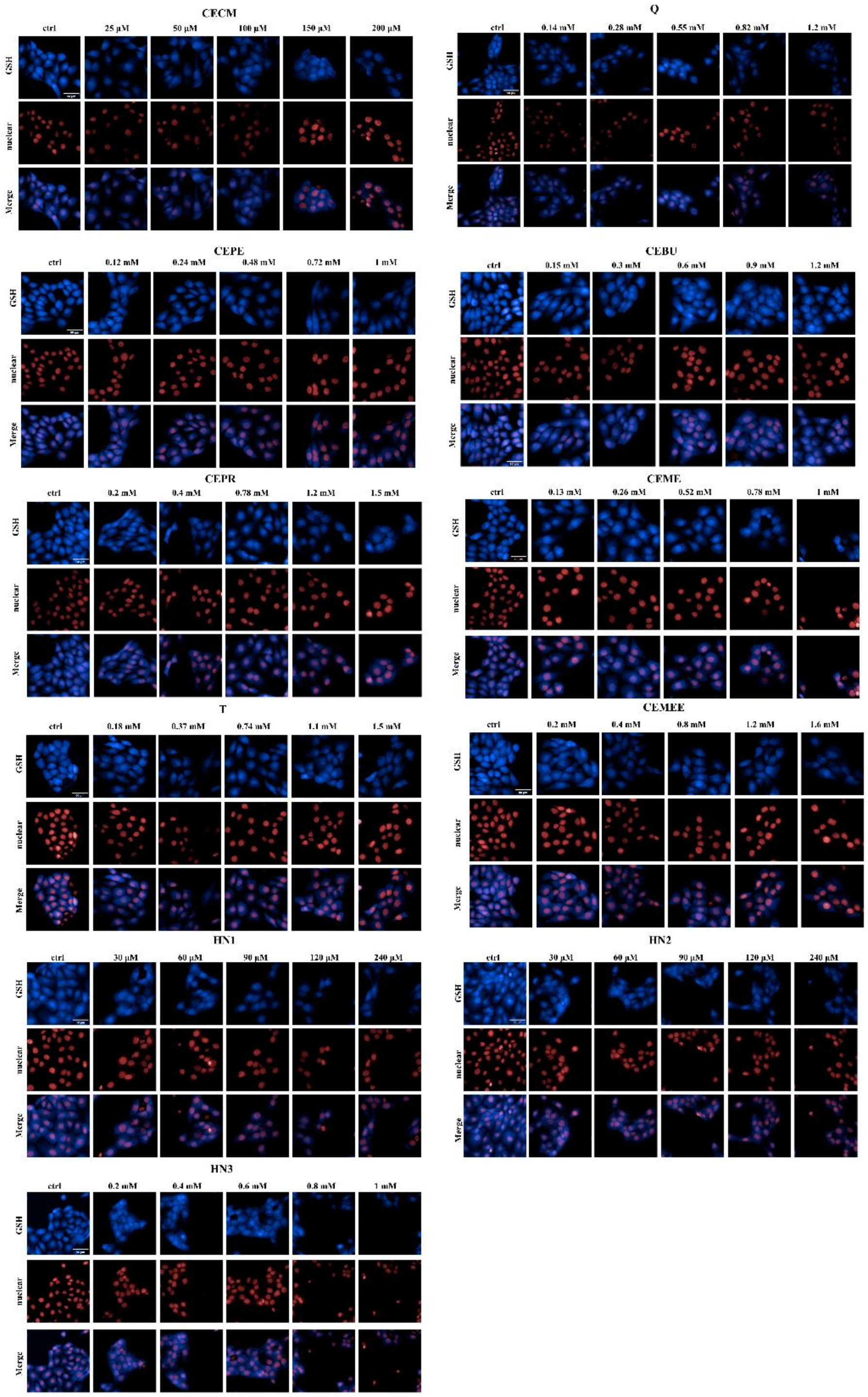

Figure S3 High-content cell imaging of GSH depletion induced by a series of alkylating agents in HaCaT cells in a dose-dependent manner.

The picture can't be displayed.

Figure S4 High-content cell imaging of GSH depletion induced by a series of alkylating agents in HaCaT cells in a time-dependent manner.
